# Chemical Characterization and In Vivo Safety Assessment of Aqueous Extract From *Anacardium occidentale* L. (Anacardiaceae) Leaves

**DOI:** 10.1002/cbdv.71528

**Published:** 2026-07-26

**Authors:** Elinado Francisco de Lima Bento, Caio Everton Nunes da Silva, Talita Giselly dos Santos Souza, Camylla Janiele Lucas Tenório, Natanael Teles Ramos de Lima, Cristiano Aparecido Chagas, José Maria Barbosa Filho, Magda Rhayanny Assunção Ferreira, Luiz Alberto Lira Soares, Samuel Paulo Cibulski, Alisson Macário de Oliveira, Thiago Henrique Napoleão

**Affiliations:** ^1^ Departamento de Bioquímica Centro de Biociências Universidade Federal de Pernambuco Recife Pernambuco Brazil; ^2^ Centro Acadêmico de Vitória Universidade Federal de Pernambuco Vitória de Santo Antão Pernambuco Brazil; ^3^ Departamento de Farmácia Centro de Ciências da Saúde Universidade Federal de Pernambuco Recife Pernambuco Brazil; ^4^ Departamento de Farmácia Universidade Federal da Paraíba João Pessoa Paraíba Brazil; ^5^ Programa de Pós‐graduação em Ciências Farmacêuticas Universidade Estadual da Paraíba Campina Grande Paraíba Brazil

**Keywords:** genotoxicity, lectins, phenolic compounds, tannins, toxicological safety

## Abstract

*Anacardium occidentale* L. leaves are used in traditional medicine for allergies, pain, and diabetes, for example; however, the safety and composition of aqueous preparations remain insufficiently characterized. This study aimed to evaluate the phytochemical profile, acute toxicity, and genotoxicity of an aqueous extract from leaves of this plant (AoLE). AoLE contained total phenolic content of 266.1 mg pyrogallol equivalents/g dry weight (DW), total tannin content of 32.2 mg tannic acid equivalents/g DW, 14.58 mg/mL proteins, and lectin activity. HPLC analysis revealed gallic acid and rutin presence. LC‐ESI‐MS identified 13 flavonoids, 6 phenolic acids, and 4 gallotannin derivatives. For toxicological evaluation, mice received a single oral dose of 2000 mg/kg. No mortality, behavioral alterations, or changes in body weight, food/water intake, hematological parameters, or biochemical markers were observed over 14 days. Organ weights and histology of the liver, kidneys, and spleen remained normal, indicating an LD_50_ > 2000 mg/kg. Genotoxicity assays showed no increase in micronucleate cells or DNA damage in the comet assay. The presence of polyphenols, tannins, flavonoids, and lectins, together with no evidence of acute toxicity under the tested conditions, supports the development of AoLE as a standardized phytopharmaceutical. Future studies should investigate chronic toxicity and long‐term stability to advance preclinical and clinical applications.

## Graphical abstract

*Anacardium occidentale* leaf aqueous extract showed rich polyphenolic composition, including flavonoids, tannins, and phenolic acids. Acute toxicity in mice revealed no mortality or adverse effects, with LD_50_ >2000 mg/kg and GHS Class 5. Genotoxicity assays were negative, supporting safety and potential phytopharmaceutical development, pending chronic and efficacy studies future research.

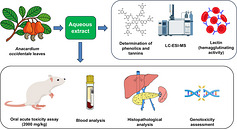

## 1 Introduction

Medicinal plants synthesize bioactive compounds capable of beneficially influencing physiological processes, making them important sources of therapeutically relevant substances. Their widespread use is attributed to availability and low cost, inclusion in everyday diets, and the general perception that plant‐based remedies are safer than conventional pharmaceuticals [1, 2]. However, although medicinal plants have demonstrated beneficial effects worldwide, they may also cause adverse effects. These undesirable effects are particularly associated with plant‐based herbal products that have not been adequately evaluated for toxicity and are insufficiently regulated [3]. Therefore, the characterization of herbal preparations and the assessment of their toxicological profiles are essential for improving health and the quality of life of individuals who use them.

*Anacardium occidentale* L. (Anacardiaceae), popularly known as the cashew tree, is a plant native to northeastern Brazil. It is a medium‐sized plant and is used in forest restoration, the timber industry, the production of oils and resins, and as a food source through its fruits and pseudofruits [4]. In traditional medicine, its leaves, bark, stem, and roots have been used to treat illnesses, including allergies, coughs, stomach pain, diarrhea, skin infections, and diabetes mellitus [5, 6]. Studies have demonstrated that *A. occidentale* leaves possess antioxidant [7], antiulcerogenic [8], hypoglycemic [9], antimicrobial [10], anti‐inflammatory [11], and antihypertensive [12] activities. It was also reported that the bark of *A. occidentale* contains lectins [13], a class of carbohydrate‐binding proteins with antimicrobial [14, 15], insecticidal [16], and pharmacological [17] activities.

One of the essential criteria for the development of phytopharmaceuticals is the evaluation of their potential toxicity and safety, as well as comprehensive phytochemical characterization. The toxicity assessment of *A. occidentale* leaf extracts has been previously documented in the literature. Konan et al. [18] evaluated the oral acute toxicity (2000 mg/kg), 30‐day subacute toxicity (400, 700, and 1000 mg/kg), and genotoxicity (2000 mg/kg) of an ethanolic leaf extract in Wistar rats and reported a favorable safety profile in all assays. Tédong et al. [19] reported that a hexane leaf extract did not induce toxic effects when orally administered as single doses of 2000 and 6000 mg/kg; however, an LD_50_ of 16,000 mg/kg was determined, and signs such as asthenia, anorexia, diarrhea, and syncope were observed at doses ≥10,000 mg/kg. In addition, in an 8‐week oral subchronic study, the same hexane extract (2000–14,000 mg/kg) reduced food intake and weight gain, induced behavioral changes, and caused liver and kidney lesions. In turn, Konan and Bacchi [8] reported no signs of acute toxicity in Swiss mice orally treated with a hexane leaf extract at 2000 mg/kg. Also, a hydromethanolic extract from leaves did not cause acute toxicity and morphological changes in organs of male chicks [20].

Although previous studies have investigated the toxicity of organic extracts and assessed the pharmacological activities of *A. occidentale* leaves, a comprehensive evaluation of the chemical profile and safety of aqueous leaf preparations, closely reflecting traditional use as teas or infusions, has not yet been conducted. To address this issue, the present study comprehensively characterized an aqueous extract of *A. occidentale* leaves (AoLE) for the presence of secondary metabolites and lectins, as well as evaluated its acute toxicity (biochemical and hematological parameters, histopathological alterations) and genotoxicity in Swiss mice (*Mus musculus*).

## 2 Materials and Methods

### 2.1 Plant Material

Leaves of *A. occidentale* were collected on 15 May 2023 from adult specimens located in the garden of the Department of Pharmacy at the *Universidade Federal de Pernambuco* (UFPE), Recife, Brazil (8°02′58.3″ S, 34°56′48.2″ W). Taxonomic identification was confirmed at the Herbarium UFP Geral Mariz of UFPE, where a voucher specimen (no. 90,032) is deposited (https://specieslink.net/rec/155/90032). Collection was performed under authorization no. 72024 from the *Instituto Chico Mendes de Conservação da Biodiversidade*, and the study is registered under A2087F0 in the *Sistema Nacional de Gestão do Patrimônio Genético e do Conhecimento Tradicional Associado* (SisGen). Immediately after collection, the leaves were rinsed with distilled water and dried in an oven (Fanem, São Paulo, Brazil) at 40°C for 5 days. The dried material was then ground into a powder and stored at −20 °C.

### 2.2 Preparation of Aqueous Extract

A 2^2^ factorial design was employed to prepare four different extracts by varying two factors: drug concentration (5% or 10%, w/v) and extraction method, either homogenization for 16 h or turboextraction in four cycles of 30 s with 4 min intervals. All extractions were performed using distilled water as the solvent. The extract selected for subsequent *in vivo* testing was determined based on total phenolic content, guided by Pareto charts and response surface analysis. After identifying the optimal extraction condition, the chosen *A. occidentale* leaf extract (AoLE) was prepared, freeze‐dried using a Liotop L101 lyophilizer (Liobras, São Carlos, Brazil) and stored at −20 °C. The yield was calculated according to Dhanani et al. [21] as the ratio of the mass of extract to the mass of dry matter.

### 2.3 Total Phenolic Content

Total phenolics were quantified using the Folin–Ciocalteu method. Samples were prepared at a concentration of 1 mg/mL. In a 10‐mL volumetric flask, 250 µL of the sample, 500 µL of Folin–Ciocalteu reagent, and 4 mL of distilled water were mixed, and the final volume was adjusted with 10% (w/w) sodium carbonate (Na_2_CO_3_) solution. Absorbance was measured in triplicate after 20 min at 780 nm. Total phenolic content was expressed as milligrams of pyrogallol equivalents (PGE) per g dry weight (DW).

### 2.4 Tannin Content

Initially, total phenol content was evaluated in 96‐well microplates following the method described by Margraf et al. [22]. Briefly, 20 µL of the sample in distilled water (0.2 mg/mL) was mixed with 100 µL of 10% (v/v) Folin–Ciocalteu reagent. After 5 min, 100 µL of 7.5% (w/v) Na_2_CO_3_ was added, and the plate was shaken for 20 s. The mixture was then allowed to rest in the dark at 25°C for 60 min before absorbance was measured at 740 nm using a microplate reader. A calibration curve (*y* = 0.0677x + 0.0455; *R* ^2^ = 0.9912) was generated using tannic acid standard (0.5–10 mg/mL), and total phenol content was expressed as milligrams of tannic acid equivalents (TAE) per gram DW. Residual phenols were quantified in parallel following Amorim et al. [23]. In this method, 1 g of casein was combined with 6 mL of the sample and 12 mL of distilled water aiming to precipitate tannins bound to casein. After stirring for 3 h, the mixture was filtered to remove the casein, and the volume was adjusted to 25 mL. Phenol content in the tannin‐free filtrate was then measured as described above. Finally, total protein‐precipitable tannins content was calculated as the difference between total phenols and residual phenols.

### 2.5 High Performance Liquid Chromatography (HPLC)

Chemical profiling of AoLE was conducted using high‐performance liquid chromatography (HPLC) on an Ultimate 3000 system (Thermo Fisher Scientific, Waltham, MA, USA) equipped with a photodiode array detector (DAD). The system included a binary pump (HPG‐3 × 00RS), degasser, and an autosampler with a 20 µL injection loop (ACC‐3000). Detection was performed at 270 and 350 nm. Separations were achieved on a C18 analytical column (250 mm × 4.6 mm i.d., 5 µm; Supelco Inc., Bellefonte, PA, USA) with a C18 guard column (4 mm × 3.9 µm; Phenomenex, Torrance, CA, USA) at a controlled temperature of 24 ± 1°C. The mobile phase consisted of 0.05% trifluoroacetic acid in ultrapure water (solvent A) and methanol (solvent B), delivered at a flow rate of 0.7 mL/min. The gradient program was as follows: 0–10 min, 15%–25% B; 10–15 min, 25% B; 15–20 min, 25%–40% B; 20–25 min, 40%–80% B; 30–32 min, 80% B; 32–34 min, 80%–15% B; and 34–36 min, 15% B. Rutin and gallic acid were used as reference standards. All samples were analyzed in triplicate, and chromatographic data were processed using Chromeleon 6.8 software (Dionex/Thermo Fisher Scientific).

### 2.6 Phytochemical Analysis by LC‐ESI‐MS

Phytochemical profiling of the AoLE was also conducted using liquid chromatography coupled to electrospray ionization mass spectrometry (LC‐ESI‐MS). Chromatographic separation was performed on an HPLC system (Shimadzu, Kyoto, Japan) equipped with a LC‐20AD solvent pump (600 µL/min), DGU‐20A5 online degasser, CBM‐20A system controller, and SPD‐M20A diode array detector (190–800 nm). A GIST C18 column (250 × 4.6 mm, 5 µm, 100 Å; Shimadzu) was used for separation, and 20 µL of a 1 mg/mL extract solution was injected via an autosampler (SIL‐20A). The mobile phase consisted of 0.1% formic acid in water (solvent A) and methanol (solvent B), and a linear gradient from 5% to 100% B over 60 min was applied to elute analytes. Mass spectrometric detection was performed on an Amazon X (Bruker Daltonics, Billerica, MA, USA) equipped with an electrospray ionization (ESI) source operating in negative mode. The following parameters were set: capillary voltage 4.5 kV, end plate offset 500 V, nebulizer pressure 4.0 bar, nitrogen drying gas at 8 mL/min, and a source temperature of 200°C. Collision‐induced dissociation (CID) was carried out in auto‐MS/MS mode, using advanced resolution for both MS and MS/MS acquisitions. Spectra were recorded over an *m*/*z* range of 50–1,500 at intervals of 2 s.

### 2.7 Protein Quantification

Protein concentration was determined according to Lowry et al. [24] using a bovine serum albumin (BSA) standard curve ranging from 31.25 to 500 µg/mL. Samples (0.2 mL) were incubated for 10 min with 1 mL of an alkaline copper solution [1 mL of 0.5% (w/v) copper sulfate in 1% (w/v) sodium citrate] and 50 mL of 2% (w/v) Na_2_CO_3_ in 0.1% (w/v) sodium hydroxide. After incubation at 25°C, 0.1 mL of Folin–Ciocalteu reagent diluted 1:1 with water was added. Following 30 min of reaction, absorbance was measured at 720 nm.

### 2.8 Evaluation of Hemagglutinating Activity

The presence of lectins in AoLE was assessed using the hemagglutinating activity assay in microtiter plates, following Procópio et al. [25]. Briefly, 50 µL of the sample was serially diluted in 0.15 M NaCl, and 50 µL of a 2.5% (v/v) suspension of glutaraldehyde‐fixed [26] rabbit erythrocytes was added. After 45 min, erythrocyte precipitation was inspected visually. Hemagglutinating activity (titer^−1^) was defined as the inverse of the highest dilution that caused complete hemagglutination. Specific hemagglutinating activity was expressed as the ratio of the titer to the protein content (mg). Rabbit erythrocytes were collected with approval from the Ethics Committee on Animal Experimentation of UFPE (process 23076.033782/2015–70). A hemagglutinating activity inhibition assay was performed to confirm the lectin presence. The extract was incubated for 15 min at 28°C with 0.2 M solutions of glucose, fructose, maltose, galactose, N‐acetylglucosamine, or methyl mannopyranoside, or with 0.5 mg/mL of casein, azocasein, or albumin, prior to determination of hemagglutinating activity.

### 2.9 Toxicological Assessment

#### 2.9.1 Animals

Female Swiss mice (6–8 weeks, 25–30 g) were obtained from the Keizo Asami Institute (iLIKA) at UFPE and maintained under standard laboratory conditions, including a 12 h light/dark cycle and a temperature of 22°C. Animals had free access to water and food (Labina, Purina, Brazil). All experimental procedures were conducted in accordance with the guidelines approved by the Ethics Committee for the Use of Animals (CEUA) of UFPE, under protocol number 136/2022.

#### 2.9.2 Acute Toxicity Assay

The experiment was conducted following the Test Guideline 423 of OECD [27], using three animals per group. To achieve a final sample size of six per group and improve statistical power, the experiment was repeated once. The control group received a single oral dose of saline solution (0.15 M NaCl), while the test group administered a single dose of AoLE at 2000 mg/kg. Animals were observed closely for the first four hours post‐administration for behavioral signs of toxicity, as described by Brito et al. [28]. Subsequently, they were monitored for 14 days, during which body weight, water and food intake, and individual behaviors were recorded. On day 14, animals were anesthetized intraperitoneally with ketamine (100 mg/kg) and xylazine (10 mg/kg) [29], and blood was collected via cardiac puncture for biochemical and hematological analyses. Liver, spleen, and kidneys were harvested for histological examination and weight.

#### 2.9.3 Biochemical and Hematological Analysis

Biochemical analyses included measurement of blood levels of albumin (ALB), alanine aminotransferase (ALT), aspartate aminotransferase (AST), alkaline phosphatase (ALP), bilirubin (BIL), gamma‐glutamyl transferase (GGT), total protein (TP), urea (UR), creatinine (CRE), total cholesterol (TC), triglycerides (TG), high‐density lipoprotein cholesterol (HDL‐c), low‐density lipoprotein cholesterol (LDL‐c), and very low‐density lipoprotein (VLDL), using commercial kits (Labtest Diagnóstica, Lagoa Santa, Brazil). Hematological evaluations included red blood cell count (RBC), hematocrit (HCT), hemoglobin (HB), mean corpuscular volume (MCV), mean corpuscular hemoglobin (MCH), mean corpuscular hemoglobin concentration (MCHC), platelet count (PLT), white blood cell count (WBC), and differential counts for segmented neutrophils (SEG), lymphocytes (LYM), monocytes (MON), basophils (BAS), and eosinophils (EOS).

#### 2.9.4 Histological Analysis

For histological analysis, organ samples were fixed in 10% (v/v) buffered formalin, dehydrated through a graded ethanol series (70%–100%), cleared in xylene, and embedded in paraffin. Sections of 5 µm thickness were cut, stained with hematoxylin and eosin, and mounted on coverslips using Entellan resin (Merck, Darmstadt, Germany) [30]. Slides were examined using a Motic BA200 microscope coupled to a Moticam 1000 digital camera (1.3 MP; Motic Incorporation Ltd., Kowloon, Hong Kong).

### 2.10 Assessment of In Vivo Genotoxicity

Groups of female mice (*n* = 5) received either oral saline or AoLE at 2000 mg/kg body weight. For the comet assay, blood was collected from the tail vein 6 h after treatment [31]. Blood cells were isolated, suspended in buffer, and subjected to electrophoresis following the protocols of Singh et al. [32] and Tice et al. [33]. Slides were subsequently stained with ethidium bromide (79.25 µM) and examined under a fluorescence microscope. DNA damage was quantified by assessing the comet tail, with cells scored from 0 (undamaged) to 4 (maximal damage).

For the micronucleus assay, the method of Hayashi et al. [34] was applied using the same animals. Blood was collected 48 h post‐treatment into anticoagulant‐containing tubes. For each animal, 10 µL of blood was placed on two slides pre‐stained with acridine orange, and micronuclei were scored under a fluorescence microscope. A total of 2000 polychromatic erythrocytes (PCE) per animal were counted by a single observer using a blinded analysis.

### 2.11 Statistical Analysis

Results are presented as mean ± standard deviation (SD). Statistical comparisons between groups were performed using one‐way analysis of variance (ANOVA) followed by Bonferroni's post hoc test. Comet and micronucleus assay data were analyzed using ANOVA with Tukey's post hoc test. All analyses were conducted using GraphPad Prism Software (La Jolla, CA, USA), and differences were considered statistically significant at *p* < 0.05.

## 3 Results and Discussion

An essential step in developing *A. occidentale*‐based phytopharmaceuticals is evaluating their safety and potential toxicity, along with comprehensive phytochemical characterization. Here, we characterized an aqueous leaf extract (AoLE) and provided a detailed chemical characterization and acute toxicity profile.

**FIGURE 1:**
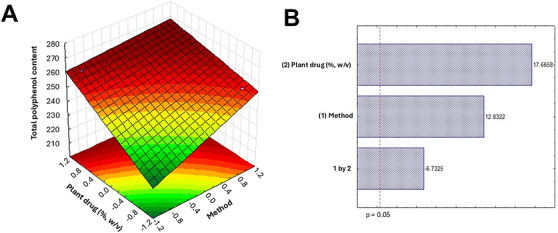
Evaluation of the influence of plant drug concentration (5 or 10%, w/v) and the method (maceration of turboextraction) used to obtain the aqueous extract from *Anacardium occidentale* leaves. The coded variables can be seen in Table 1. Response surface (A) and Pareto chart (B) for total phenolic content are shown.

Initially, four different extracts were obtained by varying the drug concentration (5% or 10%, w/v) and the extraction method (maceration or turboextraction). Table 1 shows that the extract prepared from leaf powder at a concentration of 10% (w/v) using the turboextraction method exhibited the highest total phenolic content (266.1 ± 0.80 mg PGE/g DW). The response surface plot (Figure 1A) and the Pareto chart (Figure 1B) indicated a positive influence of both the extraction method (+12.83) and drug concentration (+17.66), confirming that higher responses were obtained when the turboextraction method and a 10% concentration were employed. Therefore, this extract was selected for further studies and was designated AoLE. The yield of AoLE was 24.6 ± 0.82%, which is comparable to that reported by Nugroho et al. [12], who obtained a yield of 26.72% using hydroalcoholic maceration of *A. occidentale* leaves.

**TABLE 1:** Experimental design matrix for evaluating the extraction method and plant drug amount to obtain aqueous extract from *Anacardium occidentale* leaves.

Coded variables		Natural variables		Response
Method	Plant drug	Method	Plant drug (%)	Total phenolic content (mg PGE/g DW)
−1	−1	M	5	226.8 ± 1.80
1	−1	T	5	252.0 ± 2.78
−1	1	M	10	258.2 ± 2.91
1	1	T	10	266.1 ± 0.80

M, maceration; T, turboextraction; PGE, pyrogallol equivalents; DW, dry weight; Response values are presented as mean ± standard deviation.

The higher efficiency of turboextraction compared to homogenization can be attributed to intense agitation that disrupts cell walls, increases surface contact between plant particles and solvent, shortens extraction time, and produces slight friction‐induced heating, all of which enhance compound release and minimize degradation of sensitive metabolites [35, 36, 37]. These advantages make turboextraction particularly suitable for industrial production, allowing for more concentrated and standardized extracts as well as higher productivity.

**FIGURE 2:**
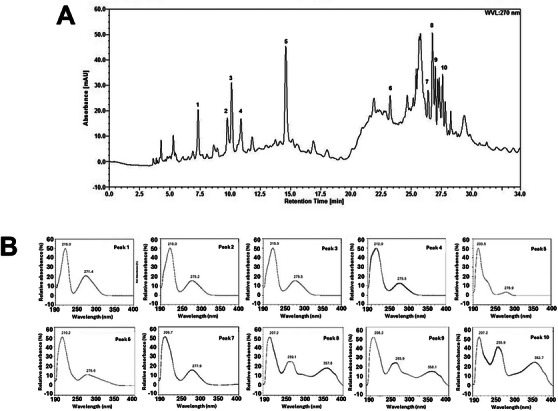
Characterization of *Anacardium occidentale* leaf extract (AoLE) by high‐performance liquid chromatography. (A) Chromatographic profile on a C18 analytical column monitored at 270 nm. (B) UV spectra of peaks 1–10 (where 1–7 was assigned to hydrolysable tannins and 8–10 was assigned to flavonoid).

The tannin content of AoLE was 32.2 mg TAE/g DW. Because the method does not allow separate identification or quantification of tannin classes, this value represents total protein‐precipitable tannins and may reflect contributions from both hydrolysable and condensed tannins. HPLC analysis enabled the characterization of the phytochemical profile of AoLE, highlighting the presence of hydrolysable tannins and flavonoids. The chromatogram (Figure 2A) showed several peaks, of which seven were assigned to the class of hydrolysable tannins [1, retention time (rt, min.) = 7.35; 2, rt = 9.76; 3, rt = 10.12; 4, rt = 10.89; 5, rt = 14.60; 6, rt = 23.24; 7, rt = 26.37] and three to the flavonoid class (8, rt = 27.17; 9, rt = 26.73; 10, rt = 27.57), as shown in Figure 2B. Based on these data, the concentration of gallic acid and rutin was calculated in AoLE, obtaining values of 0.61 ± 0.0096% (1.57%) and 0.22 ± 0.0001% (0.52%), respectively.

The presence of tannins and flavonoids in the leaf mesophyll is characteristic of the Anacardiaceae family [38]. Tannins are a hallmark of *A. occidentale* [39], and this class of compounds can be used as biomarkers in the standardization process to produce herbal medicines derived from this species. As is well known, tannins exhibit a wide range of biological activities, including antioxidant, anti‐inflammatory, antidiabetic, cardioprotective, wound‐healing, and antimicrobial effects [40]. Cashew leaf extracts containing tannins and flavonoids, as identified by thin‐layer chromatography, were reported to exhibit antihypertensive activity in isolated rat aorta [12]. However, many studies on *A. occidentale* leaf extracts did not perform chemical characterization, which hampers the association of the observed bioactivities with specific components.

LC‐ESI‐MS analysis of AoLE enabled the identification of several compounds. Within the flavonoid class, 13 components were identified. Six were flavonols: myricetin‐O‐hexoside (*m*/*z* 479) [41], quercetin‐O‐hexoside‐gallate (*m*/*z* 615) [42], quercetin‐O‐hexoside (*m*/*z* 463) [43], quercetin‐O‐pentoside (*m*/*z* 433) [44], quercetin‐O‐deoxyhexoside (*m*/*z* 477), and kaempferol‐O‐deoxyhexoside (*m*/*z* 431) [45]. Five compounds were classified as flavanols: (epi)gallocatechin→(epi)catechin (*m*/*z* 593), (epi)catechin→(epi)gallocatechin (*m*/*z* 593) [43], gallocatechin/epigallocatechin (*m*/*z* 305) [46], catechin (*m*/*z* 289), and proanthocyanidin B1 (catechin–catechin) (*m*/*z* 577) [47]. One compound was identified as a flavanonol, dihydrokaempferol‐O‐hexoside (*m*/*z* 449) [48], and one as a flavanone, naringenin‐6,8‐di‐C‐hexoside (*m*/*z* 595) [49, 50]. Quercetin‐O‐hexoside has previously been reported in other species of the Anacardiaceae family, and studies indicate that it contributes significantly to antioxidant activity as assessed by the DPPH method [44]. Naringenin‐6,8‐di‐C‐hexoside and dihydrokaempferol‐O‐hexoside have also been reported in leaf tissues, with the latter exhibiting notable antioxidant activity [48, 49, 50].

Six components belonging to the phenolic acid group were identified: gallic acid‐O‐hexoside (*m*/*z* 331) [51], gallic acid (*m*/*z* 169) [52], methyl gallate‐O‐hexoside (*m*/*z* 345) [53], hydroxycinnamic acid–gallic acyl‐O‐hexoside (*m*/*z* 477) [54], dihydroxybenzoic acid‐O‐hexoside (*m*/*z* 315) [55], and protocatechuic acid‐O‐glucuronide (*m*/*z* 329) [56]. These gallic acid derivatives are widely distributed among plant species and tissues and have been reported to exhibit antioxidant activity. In addition, one gallotannin, digalloyl‐O‐hexoside (*m*/*z* 465) [43], three gallotannin derivatives—galloylshikimic acid derivatives I, II, and III (*m*/*z* 325) [57]—and shikimic acid (*m*/*z* 173) [58, 59] were identified.

The protein concentration in AoLE was 14.58 mg/mL. *A. occidentale* leaves contain proteins at levels ranging from 10.12% to 14.65% of their biomass [60, 61], indicating a strong potential for protein extraction using water alone as the solvent in the present study.

AoLE exhibited a specific hemagglutinating activity of 8989.84, suggesting the presence of lectins. Their occurrence was confirmed, as the hemagglutinating activity was inhibited by azocasein (1.1), casein (4.39), albumin (8.78), *N*‐acetyl glucosamine (140.47), and methyl mannopyranoside (280.94). This indicates that these carbohydrates prevented the lectins in AoLE from binding to glycoconjugates on the erythrocyte surface. Maciel et al. [13] previously purified a lectin from *A. occidentale* bark (AnocBL), whose hemagglutinating activity was inhibited by L‐arabinose and glycoproteins.

Within the Anacardiaceae family, lectins have also been isolated from the bark, heartwood, and leaves of *Myracrodruon urundeuva* Allem [62, 63] and from the leaves of *Schinus terebinthifolia* Raddi [64]. The lectins from these species have demonstrated biotechnological applications as insecticidal agents [65] and biomedical potential as antimicrobial [66], antitumor [67], anti‐inflammatory [68, 69], and psychotropic [70] agents. The identification of lectins in AoLE supports future studies aiming at the purification and characterization of these proteins from cashew leaves.

Once characterized, AoLE was evaluated for oral acute toxicity in Swiss mice. Although derived from natural sources, plant extracts may elicit adverse effects in animals. Despite their biomedical potential, some constituents can be toxic depending on the dose; for example, tannins may exhibit antinutritional effects [71], and certain lectins can cause a range of toxic effects, from minor alterations to lethality [17].

AoLE (2000 mg/kg) did not induce behavioral changes during the first 4 h after administration. Throughout the 14‐day observation period, there were no significant differences (*p* < 0.05) in daily water and food consumption or in the weight gain pattern of the animals compared to the control group (Table 2). No deaths occurred during the experimental period, indicating an LD_50_> 2000 mg/kg. Therefore, AoLE can be classified as having a low oral toxicity according to the Globally Harmonized System (GHS) of Classification and Labeling of Chemicals, corresponding to Class 5 (low or no acute toxicity) [27].

**TABLE 2:** Evaluation of body weight and average daily food and water consumption of control animals and those treated with a single oral dose of 2000 mg/kg of *Anacardium occidentale* leaf extract (AoLE), assessed during 14 days.

Parameters	Control	AoLE (2000 mg/kg)
Average weight	—	—
Initial weight (g)	25.93 ± 0.81	26.26 ± 0.73
Final weight (g)	25.96 ± 2.43	27.00 ± 3.11
Food consumption (g/day)	4.09 ± 0.71	4.7 ± 0.89
Water consumption (mL/day)	9.72 ± 0.86	8.6 ± 1.29

Food and water consumption values correspond to the total consumption of each experimental group. Values represent the mean ± standard deviation (*n* = 6). No significant differences (*p* > 0.05) were found between groups according to one‐way analysis of variance (ANOVA) followed by Bonferroni's post hoc test.

Similarly, Tédong et al. [19] reported that the hexane extract from *A. occidentale* leaves did not induce toxic effects when administered as single dose of 2000 mg/kg. These authors reported that doses ≥10,000 mg/kg caused signs such as asthenia, anorexia, diarrhea, and syncope. However, the authors did not perform chemical characterization of the extract; therefore, the toxic effects cannot be attributed to any specific compounds. Considering that pharmacological doses are much lower than those assessed and the recommendations of OECD protocol, the absence of observable toxic effects at 2000 mg/kg further indicates the low acute toxicity of AoLE under the experimental conditions.

Analysis of hematological parameters revealed no significant differences (*p* > 0.05) between animals receiving 2000 mg/kg AoLE and the control group (Table 3). Similarly, biochemical analysis (Table 4) showed no significant changes (*p* > 0.05) in markers of kidney and liver function, which are sensitive indicators of organ toxicity. Overall, the lack of alterations in these parameters suggests that AoLE does not adversely affect the physiological function of key organs or that, if any adverse effects occurred, the animals could recover from them. To confirm this, the organs were collected and examined both macroscopically and microscopically.

**TABLE 3:** Hematological parameters of control animals and those treated with a single oral dose of 2000 mg/kg of *Anacardium occidentale* leaf extract (AoLE), assessed after 14 days.

Parameters	Control	AoLE
Erythrocytes (10^6^/mm^3^)	5.30 ± 0.31	5.11 ± 0.44
Hematocrit (%)	37.54 ± 3.12	39.14 ± 3.24
Hemoglobin (g/dL)	14.52 ± 0.41	14.25 ± 0.32
Mean corpuscular volume (fL)	70.83 ± 5.88	76.59 ± 6.41
Mean corpuscular hemoglobin (pg)	34.16 ± 0.71	27.88 ± 0.41
Mean corpuscular hemoglobin concentration (g/dL)	38.67 ± 1.09	36.40 ± 8.73
Platelets (10^3^/mm^3^)	996.94 ± 98.88	944.86 ± 79.49
Leukocytes (10^3^/mm^3^)	7.94 ± 0.54	7.57 ± 0.46
Lymphocytes (%)	69.54 ± 4.09	71.01 ± 3.65
Segmented neutrophils (%)	25.97 ± 0.68	24.85 ± 0.79
Monocytes (%)	2.87 ± 0.34	3.06 ± 0.42
Basophiles (%)	0.14 ± 0.02	0.11 ± 0.04
Eosinophils (%)	1.36 ± 0.15	1.40 ± 0.14

Values represent the mean ± standard deviation (*n* = 6/group). No significant differences (*p* > 0.05) were found between groups according to one‐way analysis of variance (ANOVA) followed by Bonferroni's post hoc test.

**TABLE 4:** Biochemical blood parameters of control animals and those treated with a single oral dose of 2000 mg/kg of *Anacardium occidentale* leaf extract (AoLE), assessed after 14 days.

Parameters	Control	AoLE
Albumin (g/L)	29.54 ± 3.19	32.10 ± 3.88
Alanine aminotransferase (U/L)	47.85 ± 4.12	49.17 ± 4.23
Aspartate aminotransferase (U/L)	64.16 ± 4.10	63.04 ± 5.24
Alkaline phosphatse (U/L)	13.84 ± 0.39	13.52 ± 0.44
Bilirubin (mg/dL)	0.35 ± 0.13	0.40 ± 0.10
Gamma glutamyl transferase (U/L)	14.12 ± 0.35	14.46 ± 0.44
Total proteins (g/dL)	72.12 ± 6.04	74.83 ± 5.85
Urea (mg/dL)	31.0 ± 0.44	36.0 ± 0.55
Creatinine (mg/dL)	0.40 ± 0.03	0.43 ± 0.04
Total cholesterol (mg/dL)	81.83 ± 6.41	78.42 ± 5.79
Triglycerides (mg/dL)	90.32 ± 8.45	93.51 ± 7.39
HDL‐cholesterol (mg/dL)	36.54 ± 3.08	35.01 ± 3.16
LDL‐cholesterol (mg/dL)	28.50 ± 2.56	26.97 ± 2.49
VLDL‐cholesterol (mg/dL)	14.31 ± 1.25	14.82 ± 1.00

Values represent the mean ± standard deviation (*n* = 6/group). No significant differences (*p* > 0.05) were found between groups according to one‐way analysis of variance (ANOVA) followed by Bonferroni's post hoc test.

**FIGURE 3:**
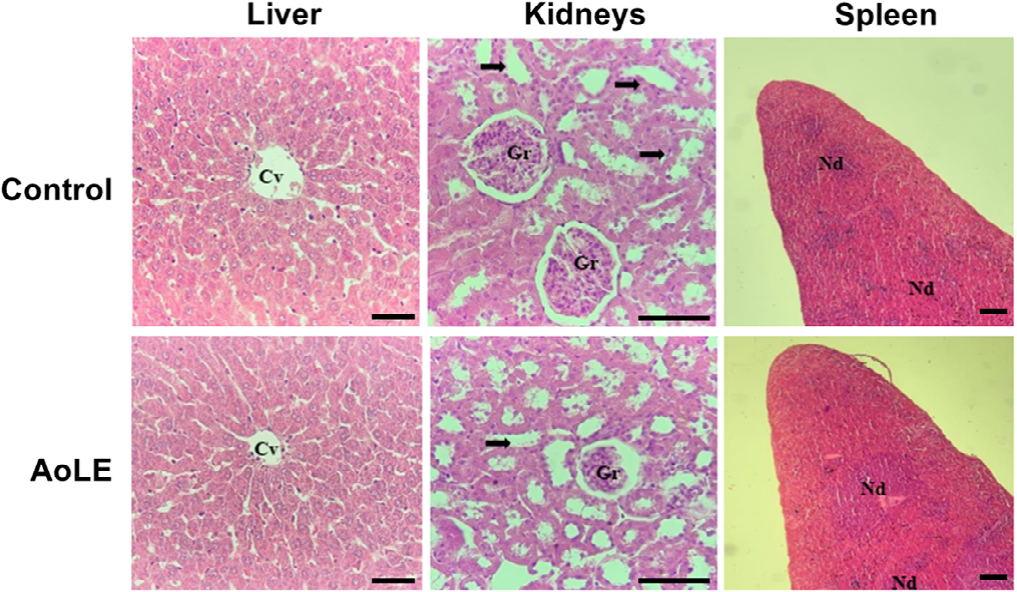
Representative photomicrographs of the liver, kidney, and spleen of female mice from the control group and those treated with a single dose of 2000 mg/kg of *Anacardium occidentale* leaf extract (AoLE). Liver: The centrilobular vein (cv) is visible in all images. Kidney: Renal glomeruli (Gr) and convoluted tubules (arrows) are well preserved and organized. Spleen: Lymph nodes (Nd) are well defined in both control and treated groups. Hematoxylin and eosin (H&E) staining was used. Scale bars: 100 µm.

No significant changes (*p* > 0.05) were observed in the organ weights of animals treated with 2000 mg/kg AoLE (Table 5), and no macroscopic alterations in color or texture were detected at the time of collection. Figure 3 shows representative histological images of the liver, kidneys, and spleen from control and AoLE‐treated mice. Livers from animals treated with AoLE exhibited clearly delineated hepatocytes, nuclei with discernible chromatin, and central lobular veins of diverse sizes with intact architecture, without signs of connective tissue fibrosis, reflecting healthy hepatic parenchyma. In the kidneys, both treated and control groups showed well‐formed glomeruli and renal convoluted tubules, indicating that AoLE did not interfere with tubular function, including reabsorption and secretion. The spleens of treated mice maintained the normal organization of white and red pulp, with typical cellular morphology and counts, and showed no indications of hyperactivation relative to controls.

**TABLE 5:** Evaluation of the relative weight of organs of control animals and those treated with a single oral dose of 2000 mg/kg of *Anacardium occidentale* leaf extract (AoLE), assessed after 14 days.

Organs	Weight (%)	
	Control	AoLE
Kidneys	0.387 ± 0.07	0.395 ± 0.04
Spleen	0.1200 ± 0.02	0.1125 ± 0.01
Liver	1.315 ± 0.22	1.307 ± 0.16

Values represent the mean ± standard deviation (*n* = 6/group). No significant differences (*p* > 0.05) were found between groups according to one‐way analysis of variance (ANOVA) followed by Bonferroni's post hoc test.

Konan et al. [18] reported the presence of tannins, flavonoids, and saponins in a hydroethanolic leaf extract that was acutely and subacutely safe in rats. Together with our results, it can be inferred that the tannins and lectins present in *A. occidentale* leaves do not occur at toxic levels, unlike tannin levels found in oak (*Quercus* spp.) leaves [42] and the highly toxic lectin ricin present in *Ricinus communis* L. seeds [72].

**FIGURE 4:**
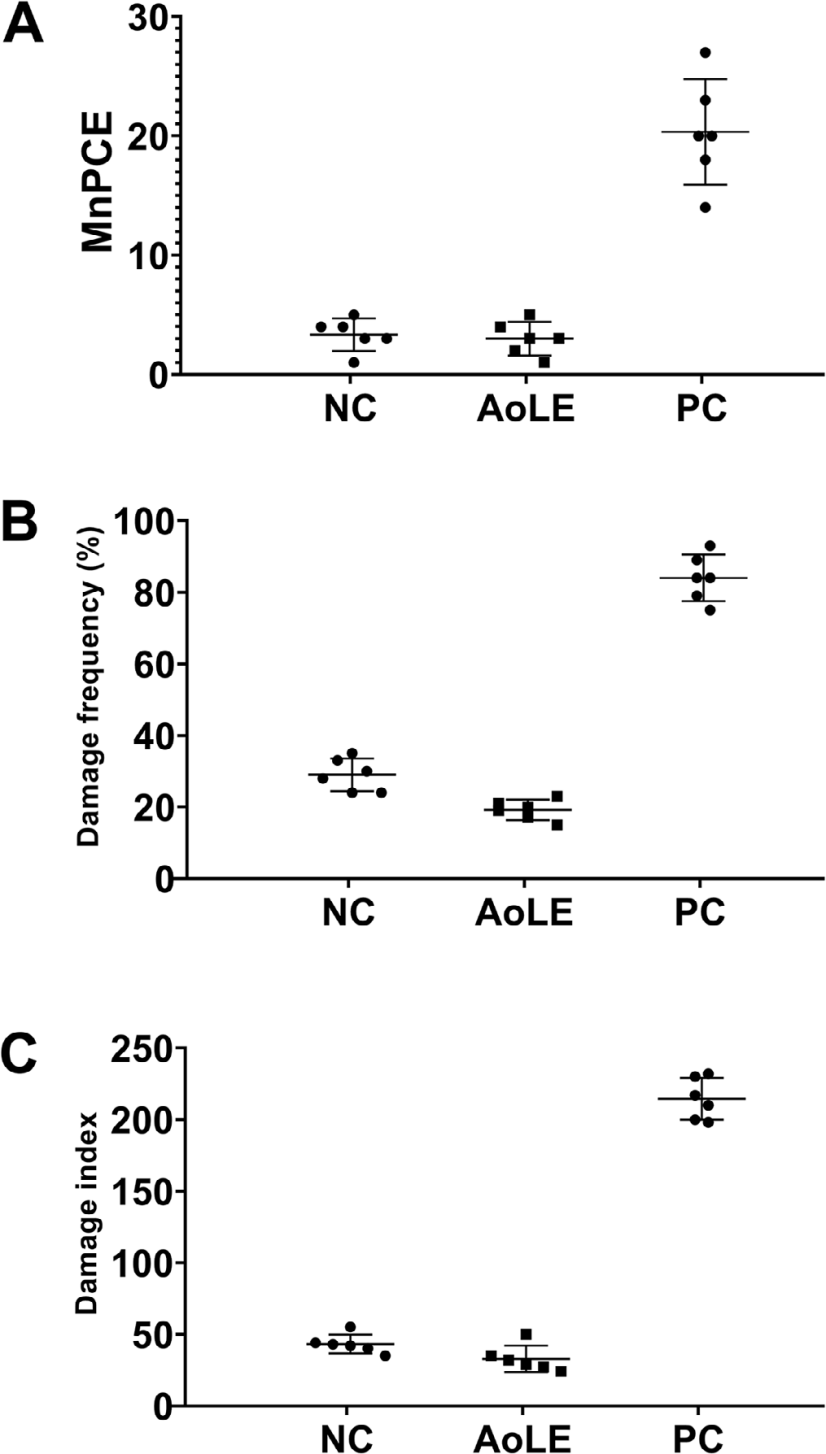
Genotoxicity assessment of *Anacardium occidentale* leaf extract (AoLE) at 2000 mg/kg in mice (*n* = 5 per group). (A) Number of micronucleated polychromatic erythrocytes (MnPCE). (B) Frequency of DNA damage in the comet assay. (C) Damage index in the comet assay. NC = negative control; PC = positive control. Error bars indicate standard deviation values. Data were analyzed using analysis of variance (ANOVA) with Tukey's post hoc test.

In the genotoxicity assessment, the micronucleus test showed no significant difference (*p* = 0.85) in the number of micronucleate PCE between the negative control and AoLE‐treated group (Figure 4A), indicating no mutagenic activity. Similarly, the comet assay revealed no significant differences in either the damage index (*p* = 0.082, Figure 4B) or damage frequency (*p* = 0.0625, Figure 4C), suggesting that acute treatment with 2000 mg/kg AoLE does not induce genotoxic effects. These findings are consistent with the absence of genotoxic effects detected in ethanolic extract from *A. occidentale* leaves [18].

The results obtained here for AoLE provide a foundation for future pharmaceutical development, particularly in standardization, safe dosing, stability, and therapeutic potential. Characterization of bioactives, including gallic acid, rutin, flavonoids, tannins, lectins, and proteins, establishes reliable chemical markers for batch‐to‐batch consistency and quality control. Acute toxicity and genotoxicity assays indicated no treatment‐related adverse effects under the experimental conditions employed, supporting further pharmacological evaluation and oral formulation development. The solubility and stability of key bioactives enable aqueous formulations, while long‐term stability under varying pH, temperature, and storage conditions should be assessed in future studies.

## 4 Conclusion

AoLE is a rich source of bioactive compounds, including polyphenols, flavonoids, tannins, and lectins, and has demonstrated low acute oral toxicity and no genotoxicity in mice. The important levels of proteins, tannins, and lectins warrant further investigation into the biological potential of cashew leaves. Overall, the low acute toxicity under the experimental conditions, the availability of standardized biomarker data, and promising therapeutic potential provide a clear rationale for the preclinical and clinical development of modern phytopharmaceuticals, including studies on sub‐acute and chronic toxicity, long‐term stability, and pharmacological efficacy.

## Author Contributions

**Elinado Francisco de Lima Bento**: conceptualization, methodology, data curation, formal analysis, investigation, visualization, writing – original draft. **Caio Everton Nunes da Silva**: investigation. **Talita Giselly dos Santos Souza**: methodology, data curation, formal analysis, investigation. **Camylla Janiele Lucas Tenório**: data curation, formal analysis, investigation, visualization, validation. **Natanael Teles Ramos de Lima**: methodology, formal analysis, investigation, resources, funding acquisition, validation. **Cristiano Aparecido Chagas**: methodology, data curation, formal analysis, resources, funding acquisition. **José Maria Barbosa Filho**: methodology, resources, funding acquisition. **Magda Rhayanny Assunção Ferreira**: methodology, data curation, formal analysis, visualization, resources, funding acquisition, validation. **Luiz Alberto Lira Soares**: methodology, data curation, formal analysis, visualization, resources, funding acquisition, validation. **Samuel Paulo Cibulski**: methodology, data curation, formal analysis, investigation, validation. **Alisson Macário de Oliveira**: conceptualization, methodology, data curation, formal analysis, investigation, visualization, writing – review and editing, supervision, resources, funding acquisition, validation. **Thiago Henrique Napoleão**: conceptualization, methodology, data curation, formal analysis, visualization, writing – review and editing, supervision, project administration, resources, funding acquisition, validation.

## Conflicts of Interest

The authors declare no conflicts of interest.

## Data Availability

The data that support the findings of this study are available from the corresponding author upon reasonable request.

## References

[1] A. S. van Wyk and G. Prinsloo , “Health, Safety and Quality Concerns of Plant‐Based Traditional Medicines and Herbal Remedies,” South African Journal of Botany 133 (2020): 54–62, 10.1016/j.sajb.2020.06.031.

[2] M. Manisha , R. Babu , A. M. Begam , K. Shakya Chahal , and A. Ashok Harale , “Medicinal Plants and Traditional Uses and Modern Applications,” Journal of Neonatal Surgery 14 (2025): 162–175, 10.52783/jns.v14.2210.

[3] T. B. Aliu , F. E. Obun , H. F. Raji , and K. O. Badmus , “Safety Evaluation and Concerns of Natural Products in Traditional Medicine,” AROC in Pharmaceutical and Biotechnology 5 (2025): 09–17, 10.53858/arocpb05010917.

[4] J. C. Farias , I. R. Vieira , S. J. Mayo , and I. M. Andrade , ““Wild Cashew Brings Many Benefits and Even Beauty”: Use and Extraction of *Anacardium occidentale* L. (cajuí) by Communities in the Parnaíba River Delta, Northeast Brazil,” Desenvolvimento e Meio Ambiente 62 (2023): 692–707, 10.5380/dma.v62i0.82153.

[5] T. K. Lim , “ *Anacardium occidentale* ,” in Edible Medicinal and Non‐Medicinal Plants (Springer, 2012), 10.1007/978-90-481-8661-7.

[6] M. H. Shahrajabian and W. Sun , “The Important Nutritional and Wonderful Health Benefits of Cashew (*Anacardium occidentale* L.)",” Natural Products Journal 13 (2023): 2–10.

[7] V. Ukwenya , O. Ashaolu , D. Adeyemi , et al., “Evaluation of Antioxidant Potential of Methanolic Leaf Extract of *Anacardium occidentale* (Linn) on the Testes of Streptozotocin‐Induced Diabetic Wistar Rats",” European Journal of Anatomy 17 (2013): 72–81.

[8] N. A. Konan and E. M. Bacchi , “Antiulcerogenic Effect and Acute Toxicity of a Hydroethanolic Extract From the Cashew (*Anacardium occidentale* L.) Leaves,” Journal of Ethnopharmacology 112 (2007): 237–242, 10.1016/j.jep.2007.03.003. 17499463

[9] S. D. Sokeng , D. Lontsi , P. F. Moundipa , H. B. Jatsa , P. Watcho , and P. Kamtchouing , “Hypoglycemic Effect of *Anacardium occidentale* L. Methanol Extract and Fractions on Streptozotocin‐Induced Diabetic Rats,” Global Journal of Pharmacology 1 (2007): 1–5.

[10] G. Anand , M. Ravinanthan , R. Basaviah , and A. V. Shetty , “In Vitro Antimicrobial and Cytotoxic Effects of *Anacardium occidentale* and *Mangifera Indica* in Oral Care",” Journal of Pharmacy & Bioallied Sciences 7 (2015): 69–74. 25709341 10.4103/0975-7406.148780 PMC4333632

[11] N. C. Souza , J. M. Oliveira , M. D. S. Morrone , et al., “Antioxidant and Anti‐Inflammatory Properties of *Anacardium occidentale* Leaf Extract",” Evidence‐Based Complementary and Alternative Medicine 2017 (2017): 2787308. 28904552 10.1155/2017/2787308 PMC5585653

[12] A. E. Nugroho , A. Malik , and S. Pramono , “Total Phenolic and Flavonoid Contents, and In Vitro Antihypertension Activity of Purified Extract of Indonesian Cashew Leaves (*Anacardium occidentale* L.),” International Food Research Journal 20 (2013): 299–305.

[13] M. I. S. Maciel , M. D. S. de Mendonça Cavalcanti , T. H. Napoleão , P. M. G. Paiva , M. T. J. de Almeida Catanho , and L. C. B. B. Coelho , “ *Anacardium occidentale* Bark Lectin: Purification, Immobilization as an Affinity Model and Influence in the Uptake of Technetium‐99 M by Rat Adipocytes,” Applied Biochemistry and Biotechnology 168 (2012): 580–591, 10.1007/s12010-012-9798-1. 22798188

[14] T. H. Napoleão , T. L. D. S. Lira , E. V. Pontual , G. R. S. Ferreira , and P. M. da Silva , “Lectins as Natural Antibiofilm Agents in the Fight Against Antibiotic Resistance: A Review,” Molecules 30 (2025): 3395, 10.3390/molecules30163395. 40871548 PMC12388734

[15] G. R. S. Ferreira , T. L. S. Lira , and T. H. Napoleão , “Targeting Yeast Pathogens With Lectins: A Narrative Review From Mechanistic Insights to the Need for Addressing Translational Challenges,” Biomedicines 14 (2026): 105, 10.3390/biomedicines14010105. 41595640 PMC12839402

[16] T. L. da Silva Lira , C. F. de Almeida , L. A. Silveira Farias , et al., “Insecticidal Activity of Saline Extract, Protein Fraction, and Lectin (BmoLL) From *Bauhinia monandra* Kurz (Fabaceae) Leaves on *Sitophilus zeamais* Motschulsky, 1855 (Coleoptera: Dryophthoridae),” Biocatalysis and Agricultural Biotechnology 71 (2026): 103893, 10.1016/j.bcab.2025.103893.

[17] A. O. Marinho , M. N. B. Silva , S. P. Silva , et al., “Preclinical Risk Assessment of Plant Lectins With Pharmacological Applications: A Narrative Review,” Molecules 31 (2026): 55. 10.3390/molecules31010055 PMC12786940 41515351

[18] N. A. Konan , E. M. Bacchi , N. Lincopan , S. D. Varela , and E. A. Varanda , “Acute, Subacute Toxicity and Genotoxic Effect of a Hydroethanolic Extract of the Cashew (*Anacardium occidentale* L.),” Journal of Ethnopharmacology 110 (2007): 30–38, 10.1016/j.jep.2006.08.033. 17088034

[19] L. Tédong , P. D. Djomeni Dzeufiet , T. Dimo , et al., “Acute and Subchronic Toxicity of *Anacardium occidentale* Linn Leaves Hexane Extract in Mice,” African Journal of Traditional, Complementary and Alternative Medicines 4 (2007): 140–147. 10.4314/ajtcam.v4i2.31194 PMC2816447 20162085

[20] J. N. Omeke , A. O. Anaga , and J. A. Okoye , “Brine Shrimp Lethality and Acute Toxicity Tests of Different Hydro‐Methanol Extracts of *Anacardium occidentale* using In Vitro and In Vivo Models: A Preliminary Study,” Comparative Clinical Pathology 27 (2018): 1717–1721, 10.1007/s00580-018-2798-y.

[21] T. Dhanani , S. Shah , N. A. Gajbhiye , and S. Kumar , “Effect of Extraction Methods on Yield, Phytochemical Constituents and Antioxidant Activity of *Withania Somnifera* ,” Arabian Journal of Chemistry 10 (2017): S1193–S1199, 10.1016/j.arabjc.2013.02.015.

[22] T. Margraf , A. R. Karnopp , N. D. Rosso , and D. Granato , “Comparison Between Folin‐Ciocalteu and Prussian Blue Assays to Estimate the Total Phenolic Content of Juices and Teas Using 96‐Well Microplates,” Journal of Food Science 80 (2015): C2397–C2403, 10.1111/1750-3841.13077. 26448565

[23] E. L. Amorim , J. E. Nascimento , J. M. Monteiro , T. J. S. Peixoto Sobrinho , T. A. Araújo , and U. P. Albuquerque , “A Simple and Accurate Procedure for the Determination of Tannin and Flavonoid Levels and some Applications in Ethnobotany and Ethnopharmacology,” Functional Ecosystems and Communities 2 (2008): 88–94.

[24] O. H. Lowry , N. J. Rosebrough , A. L. Farr , and R. J. Randall , “Protein Measurement With the Folin Phenol Reagent,” Journal of Biological Chemistry 193 (1951): 265–275, 10.1016/S0021-9258(19)52451-6. 14907713

[25] T. F. Procópio , L. L. S. Patriota , M. C. Moura , et al., “CasuL: A New Lectin Isolated From *Calliandra Surinamensis* Leaf Pinnulae With Cytotoxicity to Cancer Cells, Antimicrobial Activity and Antibiofilm Effect,” International Journal of Biological Macromolecules 98 (2017): 419–429. 28174088 10.1016/j.ijbiomac.2017.02.019

[26] D. H. Bing , J. G. M. Weyand , and A. B. Stavitsky , “Hemagglutination With Aldehyde‐Fixed Erythrocytes for Assay of Antigens and Antibodies,” Experimental Biology and Medicine 124 (1967): 1166–1170, 10.3181/00379727-124-31953. 6024827

[27] OECD *Test No. 423: Acute Oral Toxicity—Acute Toxic Class Method*, OECD Guidelines for the Testing of Chemicals, Section 4, OECD Publishing, Paris, 2002.

[28] J. S. Brito , A. O. Marinho , L. C. B. B. Coelho , et al., “Toxicity and Antitumor Activity of the Water‐Soluble Lectin From *Moringa Oleifera* Lam. Seeds (WSMoL) in Sarcoma 180‐Bearing Mice,” Toxicon 234 (2023): 107306, 10.1016/j.toxicon.2023.107306. 37778740

[29] IACUC *Office of the Institutional Animal Care and Use Committee (IACUC). Anesthesia (Guideline). Vertebrate Animal Research*, **2026**, https://animal.research.uiowa.edu/iacuc‐guidelines‐anesthesia. Accessed: 14 June 2026.

[30] J. A. Kiernan , Histological and Histochemical Methods: Theory and Practice, 4th ed. (Scion Publishing, 2008).

[31] OECD *Test No. 489: In Vivo Mammalian Alkaline Comet Assay*, OECD Guidelines for the Testing of Chemicals, Section 4, OECD Publishing, Paris, 2016.

[32] N. P. Singh , M. T. McCoy , R. R. Tice , and E. L. Schneider , “A Simple Technique for Quantitation of Low Levels of DNA Damage in Individual Cells,” Experimental Cell Research 175 (1988): 184–191, 10.1016/0014-4827(88)90265-0. 3345800

[33] R. R. Tice , E. Agurell , D. Anderson , et al., “Single Cell Gel/Comet Assay: Guidelines for In Vitro and In Vivo Genetic Toxicology Testing,” Environmental and Molecular Mutagenesis 35 (2000): 206–221, 10.1002/(SICI)1098-2280(2000)35:3<206::AID-EM8>3.0.CO;2-J. 10737956

[34] M. Hayashi , T. Morita , Y. Kodama , T. Sofuni , and M. Ishidate , “The Micronucleus Assay With Mouse Peripheral Blood Reticulocytes Using Acridine Orange‐Coated Slides,” Mutation Research Letters 245 (1990): 245–249, 10.1016/0165-7992(90)90153-B. 1702516

[35] É. da Silva Santos , F. P. Garcia , P. M. Outuki , et al., “Optimization of Extraction Method and Evaluation of Antileishmanial Activity of Oil and Nanoemulsions of *Pterodon pubescens* Benth. Fruit Extracts,” Experimental Parasitology 170 (2016): 252–260, 10.1016/j.exppara.2016.10.004. 27725158

[36] B. J. Mano‐Sousa , B. C. Alves , F. P. Andrade , and J. M. Duarte‐Almeida , “Is Turbo‐Extraction an Efficient Method for Obtaining Cannabinoids?” Research, Society and Development 11 (2022): e450111534562, 10.33448/rsd-v11i15.34562.

[37] I. Ghenabzia , H. Hemmami , I. Ben Amor , S. Zeghoud , B. Ben Seghir , and R. Hammoudi , “Different Methods of Extraction of Bioactive Compounds and Their Effect on Biological Activity: A Review,” International Journal of Secondary Metabolite 10 (2023): 469–494, 10.21448/ijsm.1225936.

[38] A. G. Cunha , E. S. Brito , C. F. Moura , P. R. Ribeiro , and M. R. A. Miranda , “UPLC–qTOF‐MS/MS‐Based Phenolic Profile and Their Biosynthetic Enzyme Activity Used to Discriminate Between Cashew Apple (*Anacardium occidentale* L.) Maturation Stages,” Journal of Chromatography B 1051 (2017): 24–32, 10.1016/j.jchromb.2017.02.022. 28285020

[39] A. L. L. E. Reis , D. S. D. Silva , K. L. F. Silva , and D. B. D. Chagas , “Caracterização Anatômica e Histoquímica de Raízes e Folhas de Plântulas de *Anacardium occidentale* L. (Anacardiaceae),” Revista Árvore 38 (2014): 209–219, 10.1590/S0100-67622014000200001.

[40] M. Fraga‐Corral , P. Otero , J. Echave , et al., “By‐Products of Agri‐Food Industry as Tannin‐Rich Sources: A Review of Tannins′ Biological Activities and Their Potential for Valorization,” Foods 10 (2021): 137, 10.3390/foods10010137. 33440730 PMC7827785

[41] S. Affes , A. Ben Younes , D. Frikha , et al., “ESI‐MS/MS Analysis of Phenolic Compounds From *Aeonium arboreum* Leaf Extracts and Evaluation of Their Antioxidant and Antimicrobial Activities,” Molecules 26 (2021): 4338, 10.3390/molecules26144338. 34299613 PMC8306197

[42] L. Molina‐García , R. Martínez‐Expósito , M. L. F. Córdova , and E. J. Llorent‐Martínez , “Determination of the Phenolic Profile and Antioxidant Activity of Leaves and Fruits of Spanish *Quercus coccifera* ,” Journal of Chemistry 2018 (2018): 2573270.

[43] C. Li and N. P. Seeram , “Ultra‐Fast Liquid Chromatography Coupled With Electrospray Ionization Time‐of‐Flight Mass Spectrometry for the Rapid Phenolic Profiling of Red Maple (*Acer rubrum*) Leaves",” Journal of Separation Science 41 (2018): 2331–2346. 29512337 10.1002/jssc.201800037 PMC7167591

[44] A. Patient , E. Jean‐Marie , J.‐C. Robinson , et al., “Polyphenol Composition and Antioxidant Activity of *Tapirira guianensis* Aubl. (Anarcadiaceae) Leaves,” Plants 11 (2022): 326, 10.3390/plants11030326. 35161307 PMC8837918

[45] L. Barros , M. Dueñas , I. C. Ferreira , A. M. Carvalho , and C. Santos‐Buelga , “Use of HPLC–DAD–ESI/MS to Profile Phenolic Compounds in Edible Wild Greens From Portugal ",” Food Chemistry 127 (2011): 169–173.

[46] D. Escobar‐Avello , J. Lozano‐Castellón , C. Mardones , et al., “Phenolic Profile of Grape Canes: Novel Compounds Identified by LC‐ESI‐LTQ‐Orbitrap‐MS,” Molecules 24 (2019): 3763, 10.3390/molecules24203763. 31635434 PMC6832258

[47] J. Sun , X. Liu , T. Yang , J. Slovin , and P. Chen , “Profiling Polyphenols of Two Diploid Strawberry (*Fragaria vesca*) Inbred Lines Using UHPLC‐HRMS^n^",” Food Chemistry 146 (2014): 289–298. 24176345 10.1016/j.foodchem.2013.08.089 PMC3902803

[48] M. D. P. Fernández‐Poyatos , A. Ruiz‐Medina , G. Zengin , and E. J. Llorent‐Martínez , “Phenolic Characterization, Antioxidant Activity, and Enzyme Inhibitory Properties of *Berberis thunbergii* DC. Leaves: A Valuable Source of Phenolic Acids,” Molecules 24 (2019): 4171, 10.3390/molecules24224171. 31744256 PMC6891573

[49] S. Li , Z. Lin , H. Jiang , L. Tong , H. Wang , and S. Chen , “Rapid Identification and Assignation of the Active Ingredients in Fufang Banbianlian Injection Using HPLC‐DAD‐ESI‐IT‐TOF‐MS,” Journal of Chromatographic Science 54 (2016): 1225–1237, 10.1093/chromsci/bmw055. 27107094

[50] Y. Lu , S. Zhu , Y. He , et al., “Systematic Characterization of Flavonoids From *Siraitia grosvenorii* Leaf Extract Using an Integrated Strategy of High‐Speed Counter‐Current Chromatography Combined With Ultra High Performance Liquid Chromatography and Electrospray Ionization Quadrupole Time‐of‐Flight Mass Spectrometry",” Journal of Separation Science 43 (2020): 852–864. 31773887 10.1002/jssc.201900789

[51] A. K. Sandhu and L. Gu , “Antioxidant Capacity, Phenolic Content, and Profiling of Phenolic Compounds in the Seeds, Skin, and Pulp of *Vitis rotundifolia* (Muscadine Grapes) As Determined by HPLC‐DAD‐ESI‐MS^n^ ,” Journal of Agricultural and Food Chemistry 58 (2010): 4681–4692, 10.1021/jf904211q. 20334341

[52] J. H. Lee , J. V. Johnson , and S. T. Talcott , “Identification of Ellagic Acid Conjugates and Other Polyphenolics in Muscadine Grapes by HPLC‐ESI‐MS,” Journal of Agricultural and Food Chemistry 53 (2005): 6003–6010, 10.1021/jf050468r. 16028988

[53] R. B. D. A. Gomes , E. S. de Souza , N. S. Gerhardt Barraqui , et al., “Residues From the Brazilian Pepper Tree (*Schinus terebinthifolia* Raddi) Processing Industry: Chemical Profile and Antimicrobial Activity of Extracts Against Hospital Bacteria,” Industrial Crops and Products 143 (2020): 111430, 10.1016/j.indcrop.2019.05.079.

[54] H. Y. Zhao , M. X. Fan , X. Wu , et al., “Chemical Profiling of the Chinese Herb Formula Xiao‐Cheng‐Qi Decoction Using Liquid Chromatography Coupled With Electrospray Ionization Mass Spectrometry,” Journal of Chromatographic Science 51 (2013): 273–285, 10.1093/chromsci/bms138. 22977122

[55] W. Grati , S. Samet , B. Bouzayani , et al., “HESI‐MS/MS Analysis of Phenolic Compounds From *Calendula aegyptiaca* Fruits Extracts and Evaluation of Their Antioxidant Activities",” Molecules 27 (2022): 2314. 35408713 10.3390/molecules27072314 PMC9000822

[56] B. F. Zimmermann , S. G. Walch , L. N. Tinzoh , W. Stühlinger , and D. W. Lachenmeier , “Rapid UHPLC Determination of Polyphenols in Aqueous Infusions of *Salvia officinalis* L. (sage tea)",” Journal of Chromatography B 879 (2011): 2459–2464. 10.1016/j.jchromb.2011.06.038 21783434

[57] C. C. Wyrepkowski , D. Gomes da Costa , A. Sinhorin , et al., “Characterization and Quantification of the Compounds of the Ethanolic Extract From Caesalpinia Ferrea Stem Bark and Evaluation of Their Mutagenic Activity,” Molecules 19 (2014): 16039–16057, 10.3390/molecules191016039. 25299821 PMC6271747

[58] D. Fraternale , D. Ricci , G. Verardo , A. Gorassini , V. Stocchi , and P. Sestili , “Activity of *Vitis vinifera* Tendrils Extract Against Phytopathogenic Fungi,” Natural Product Communications 10 (2015): 1037–1042, 10.1177/1934578X1501000661. 26197546

[59] Z. Nurazah , “Metabolomics Unravel Differences Between Cameroon Dura and deli Dura Oil Palm (*Elaeis guineensis* Jacq.) Genetic Backgrounds Against Basal Stem Rot,” Journal of Oil Palm Research 29 (2017): 227–241, 10.21894/jopr.2017.2902.07.

[60] Y. Martínez , L. A. Tobar , H. M. Lagos , C. A. Parrado , A. M. Urquía , and M. Valdivié , “Phytobiotic Effect of *Anacardium occidentale* L. Leaves Powder on Performance, Carcass Traits, and Intestinal Characteristics in Broilers,” Brazilian Journal of Poultry Science 23 (2021): 1–10.

[61] O. D. Oloruntola , “Proximate, Phytochemical, Mineral Composition and Antioxidant Activity of *Anacardium occidentale* L. Leaf Powder,” DYSONA—Life Science 2 (2021): 39, 10.30493/dls.2021.290718.

[62] R. A. Sá , N. D. L. Santos , C. S. B. Silva , et al., “Larvicidal Activity of Lectins From *Myracrodruon urundeuva* on *Aedes aegypti* ,” Comparative Biochemistry and Physiology Part C 149 (2009): 300–306. 18761426 10.1016/j.cbpc.2008.08.004

[63] T. H. Napoleão , F. S. Gomes , T. A. Lima , et al., “Termiticidal Activity of Lectins From *Myracrodruon urundeuva* Against *Nasutitermes corniger* and Its Mechanisms",” International Biodeterioration & Biodegradation 65 (2011): 52–59.

[64] F. S. Gomes , T. F. Procópio , T. H. Napoleão , L. C. B. B. Coelho , and P. M. G. Paiva , “Antimicrobial Lectin From *Schinus terebinthifolius* Leaf",” Journal of Applied Microbiology 114 (2013): 672–679. 23190078 10.1111/jam.12086

[65] T. H. Napoleão , L. P. Albuquerque , N. D. L. Santos , et al., “Insect Midgut Structures and Molecules as Targets of Plant‐Derived Protease Inhibitors and Lectins,” Pest Management Science 75 (2019): 1212–1222, 10.1002/ps.5233. 30306668

[66] M. C. Moura , T. F. Procópio , G. R. S. Ferreira , et al., “Anti‐Staphylococcal Effects of *Myracrodruon urundeuva* Lectins on Nonresistant and Multidrug Resistant Isolates,” Journal of Applied Microbiology 130 (2021): 745–754, 10.1111/jam.14811. 32750211

[67] D. B. M. Ramos , M. T. M. F. Araújo , T. C. L. Araújo , et al., “Evaluation of Antitumor Activity and Toxicity of *Schinus Terebinthifolia* Leaf Extract and Lectin (SteLL) in Sarcoma 180‐Bearing Mice",” Journal of Ethnopharmacology 233 (2019): 148–157. 30658183 10.1016/j.jep.2019.01.011

[68] A. de Oliveira Marinho , J. Alves da Costa , A. N. Silva dos Santos , et al., “Assessment of Acute Toxicity, Genotoxicity, and Anti‐Inflammatory Activity of SteLL, a Lectin From *Schinus terebinthifolia* Raddi. Leaves, in Mice,” Journal of Ethnopharmacology 333 (2024): 118496, 10.1016/j.jep.2024.118496. 38936643

[69] A. D. O. Marinho , M. N. B. da Silva , A. K. V. Chagas , et al., “The Lectin From *Schinus terebinthifolia* Raddi Leaves (SteLL) Exhibited Anti‐Inflammatory Activity in Lipopolysaccharide‐Induced Acute Lung Injury in Mice,” International Journal of Molecular Sciences 27 (2026): 3394, 10.3390/ijms27083394. 42074038 PMC13116781

[70] B. R. F. Lima , L. L. S. Patriota , A. O. Marinho , et al., “ *Schinus terebinthifolia* Raddi. Leaf Lectin (SteLL) Demonstrates Anxiolytic and Antidepressant Effects Under Monoaminergic Deficiency Induced by Reserpine,” Plants 14 (2025): 3048. 41095189 10.3390/plants14193048 PMC12525816

[71] A. Zayed , S. Abdelkareem , N. Talaat , D. A. Dayem , and M. A. Farag , “Tannin in Foods: Classification, Dietary Sources, and Processing Strategies to Minimize Anti‐Nutrient Effects,” Food and Bioprocess Technology 18 (2025): 9221–9249, 10.1007/s11947-025-04020-3.

[72] S. Olsnes and J. V. Kozlov , “Ricin,” Toxicon 39 (2001): 1723–1728, 10.1016/S0041-0101(01)00158-1. 11595634

